# The International Medical Annual and Practitioner’s Index

**Published:** 1898-05

**Authors:** 


THE INTERNATIONAL MEDICAL ANNUAL AND PRACTITIONER'S INDEX. A Work of Reference for Modern Practitioners. Sixteenth year. New York: E. B. Treat, 241–243 W. 24th street. 1898.

The sixteenth annual volume of the Medical Annual, which has just appeared, is on the same lines which have already met with the approval of hundreds of physicians the country over. Besides reviewing the progress of medical science from year to year, special articles on subjects which require bringing up to date, and concerning which it is desirable to have direct information from those who by their special experience are in a position to afford it, are added, thereby enhancing greatly the value of the annual to the busy practitioner.

In this volume the review of therapeutic progress and dictionary of new remedies is carefully gone over by William Murrell, M. D., M. R. C. P. No great novelty in the domain of pharmacology and therapeutics has appeared during the past year. The author does not believe that the treatment of diphtheria by antitoxin is making the advance that was anticipated, partly because there is no guarantee —except in Germany—that all antitoxins are standardised in the same way. The greatest progress doubtless has been made in the applicability of the thyroid gland in practical medicine. Myxedema, obesity, some forms of insanity, skin diseases and myopathies have all been greatly benefited by this product.

An interesting article by A. D. Rockwell, of New York, follows, on electro-therapeutics. The author says, in beginning his chapter, that the value of electricity in neurasthenia is but indifferently appreciated, even by the neurologist. The value of the Röntgen rays is commented on, especially in their ability to diagnosticate renal and vesical calculi. Hypnotism and suggestion, though somewhat threadbare, are treated of by Charles Lloyd Inckey. Nothing new or original is brought out in this essay. The dictionary of new treatment in medicine and surgery takes up the major part of the volume and contains some valuable short contributions, such as The consideration of ear diseases, by J. Dundas Grant, Insanity, by James Shaw, and Obliteration of the deformity in Pott's disease, with many full-page illustrative plates, by Robert Jones and A. H. Inbly. An atlas of the bacteria pathogenic in man, by S. G. Shattock, accompanied by a practical description of the methods of isolating and examining these disease germs, is a welcome addition to the 1898 annual.

Part III. is devoted to special chapters on various topics, as Legal decisions affecting medical men and the public health, A review of the progress in sanitary science, New instruments, New books, ending with the general index, making a handsome volume of 740 pages.

To those who possess the preceding volumes this needs no introduction; to those who don't, we can cheerfully recommend the series,

